# Dynamic-range compression and contrast enhancement in swept-source optical coherence tomography systems with a frequency gain compensation amplifier

**DOI:** 10.1117/1.JBO.25.11.110502

**Published:** 2020-11-27

**Authors:** Shanshan Liang, Xinyu Li, Yao Qin, Jun Zhang

**Affiliations:** a Sun Yat-Sen University, School of Electronics and Information Technology, Guangzhou, China; b Guilin University of Electronic Technology, School of Artificial Intelligence, Guilin, China

**Keywords:** Swept-source OCT, dynamic-range compression, contrast enhancement, attenuation compensation, frequency gain compensation amplifier

## Abstract

**Significance:** Optical coherence tomography (OCT) has been widely used in clinical studies. However, the image quality of OCT decreases with increasing imaging depth since the light is rapidly attenuated in biological tissues.

**Aim:** We present a compensation approach to preserve weak high-frequency signals from deep structures and compress the dynamic range of the detected signal for superior analog-to-digital conversion and image display capability.

**Approach:** A homemade frequency gain compensation amplifier is designed and fabricated to amplify the electrical signal from a balanced photodetector and compensate for the signal attenuation in swept-source OCT (SSOCT).

**Results:** It is demonstrated in imaging various objects that this cost-efficient technique effectively enhances the contrast of the deep tissue image.

**Conclusions:** A frequency gain compensation amplifier is designed and used to compress the dynamic range of the electrical signal detected by the photodetector of an SSOCT system, which enables weak signals from deep structures to be acquired by the ADC and displayed with enhanced local contrast.

## 1 Introduction

Optical coherence tomography (OCT) is an emerging noninvasive biomedical optical imaging modality that enables high-resolution cross-sectional imaging in biological tissue by detecting backreflected/scattered light from the object.^1^ Swept-source OCT (SSOCT), the latest technology of OCT, has increasingly gained attention due to its relatively simple system structure and high imaging speed.^2^ Although SSOCT has been widely used in clinical diagnosis and research areas, there remain some obstacles that hinder its application in the clinic. Due to the light attenuation caused by scattering and absorption inside the tissue, the OCT signal intensity decays dramatically with increasing imaging depth. Hence, the deep tissue image suffers from low contrast and a lack of useful structural information. To increase the contrast and visibility of deep tissue images, numerical methods have been used to compensate for the signal decay based on the analysis of OCT signals.^3^^–^^6^ However, it is difficult for the commonly used analog-to-digital converter (ADC) of an SSOCT system to fulfil the wide range of the echo signal caused by the light attenuation in tissue.^7^^,^^8^ Therefore, the weak light signal from deep tissue might be submerged in ADC quantization noise. Despite the improved image quality of deep structures, numerical methods cannot digitally recover the weak signals embedded in the background noise.

Ultrasound imaging often uses a time gain compensation circuit to compress the wide range of the input signal into the limited dynamic range of the ADC and to improve image quality by compensating for the signal decay with depth, as the propagation time represents the depth information of the image.^9^^–^^11^ In SSOCT, the depth does not correspond to the propagation time. Instead, the structural information along the depth is encoded with the frequency of the electrical signal detected by a photodetector;^12^ i.e., the electrical frequency of the interference signal increases with increasing depths. In this paper, a homemade frequency gain compensation amplifier is designed and implemented before the ADC to compensate for the weak high-frequency SSOCT signal. With the developed amplifier, the signal from the deeper tissue layers experiences a larger gain before analog-to-digital conversion. Hence, the perception of details and the contrast are improved. This compensation method could be used in many existing SSOCT systems to increase deep tissue contrast, such as intravascular, airway, and ophthalmic imaging, and it could be helpful for tissue segmentation^13^ and boundary detection.^4^

## 2 Methods

### 2.1 OCT System Setup

**Fig. 1:**
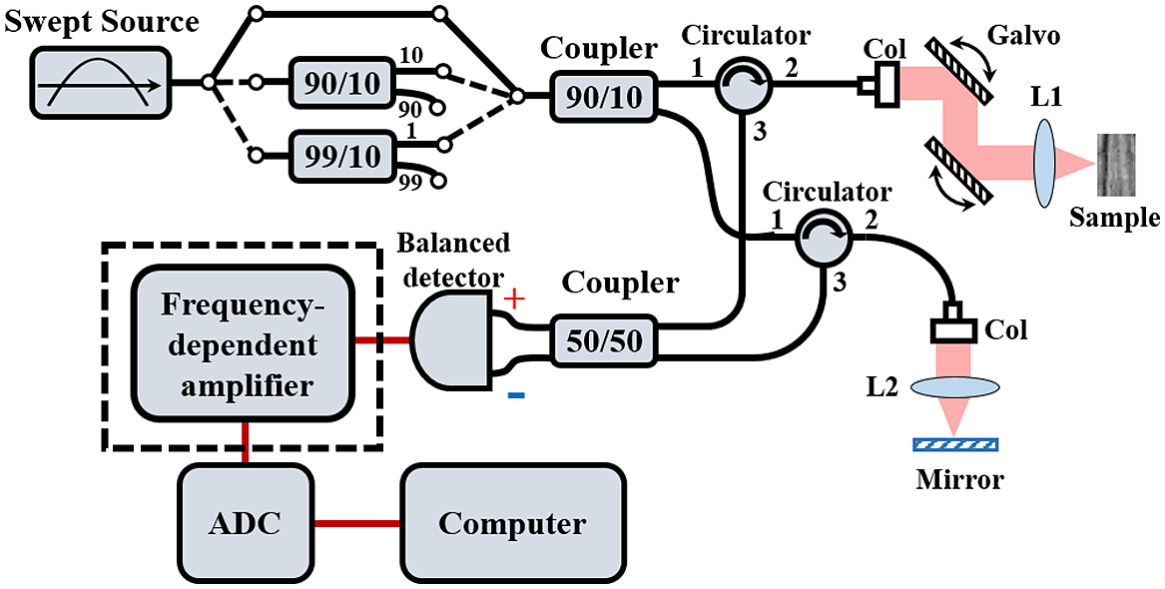
Schematic of the SSOCT system. Col, collimator; ADC, analog-to-digital converter.

The SSOCT system design is shown in Fig. 1. A swept source at 1310 nm with a bandwidth of 87 nm, a sweep frequency of 100 kHz, and an output power of 20 mW is used as the light source. The coherence length of the swept source is 8 mm, and the corresponding instantaneous linewidth is 0.093 nm. The power entering the system is adjusted by switching the optical splitter with a different split ratio. Then, a 90:10 coupler splits the input signal into the sample and the reference arm. The focus lengths of the objective in the sample arm and the reference arm are 36 and 40 mm, respectively. A 90:10 coupler splits the output light from the swept source into the sample and reference arms. In the detection arm, the interference signal detected by a balanced detector (Thorlabs, PDB 430C) is amplified by a homemade frequency gain compensation amplifier. It is recorded by an ADC (ATS9360, Alazar).

### 2.2 Frequency Gain Compensation Amplifier Design and Fabrication

**Fig. 2:**
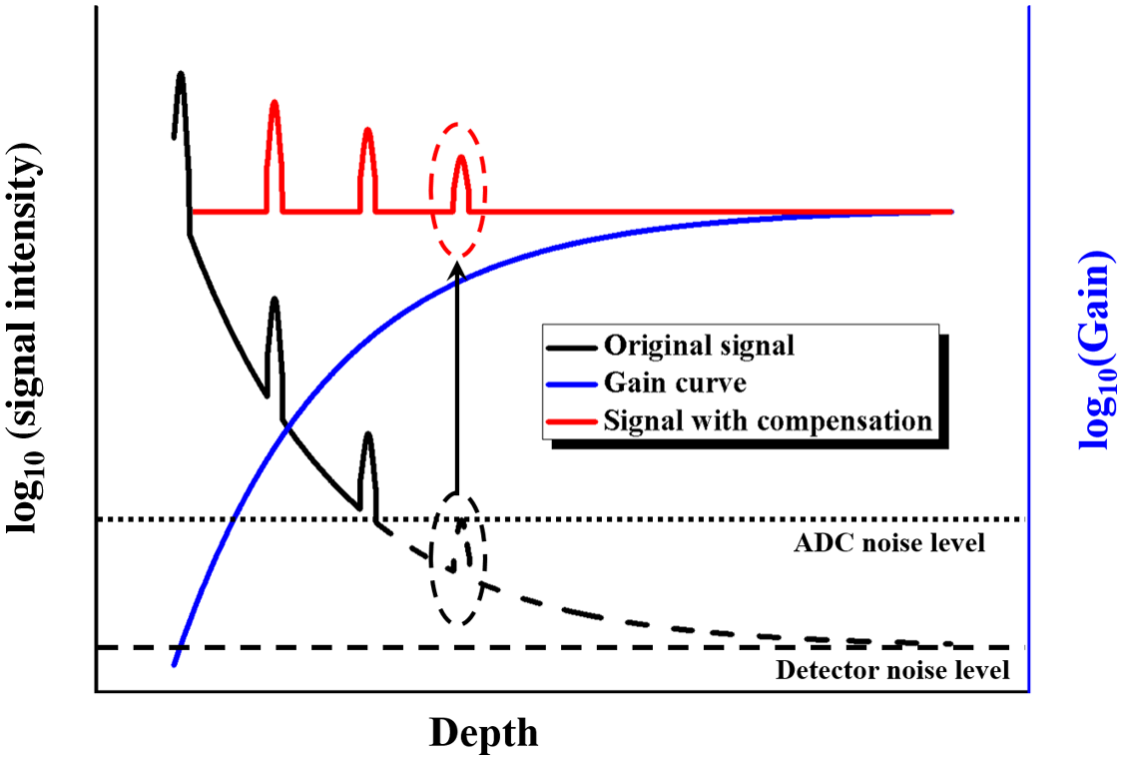
Schematic of the A-line intensity profile in biological tissue without (black curve) and with (red curve) compensation.

According to the Beer–Lambert law, the light signal exponentially decays with increasing depth,^14^ as shown in a schematic illustration of OCT A-line profiles (Fig. 2). The dashed and dotted lines in Fig. 2 represent the levels of the two dominant noise sources of the SSOCT system, which are calculated from the noise equivalent power (NEP) of the balanced photodetector and the quantization noise of the ADC, respectively. The minimal detectable signal of a balanced photodetector $N_{pd}$ is given as $N_{pd}=G\times NEP\times \sqrt{f}$, where G is the conversion gain of the photodetector and f is the measurement bandwidth. Hence, the $N_{pd}$ of our balance detector (PDB 430C, Thorlabs) in the experiment is calculated to be 0.011 mV. The quantization noise q of an N-bit ADC is calculated as $q=A/(2^{N}\times \sqrt{12})$, where A is the full-scale amplitude of the ADC. Therefore, the q of the ADC (ATS9360, Alazar) used in the experiment is 0.056 mV. Obstructed by the limited bit depth, the dynamic range of the ADC is usually much smaller than that of a balanced detector in most SSOCT systems. For example, the detector used in our experiment is capable of a wide range of ∼100  dB,^15^ which is sufficient to receive OCT signals, while the 12-bit ADC in our experiment only provides a range of 57 dB,^16^ which is usually less than the signal range, resulting in saturation effects commonly observed in OCT imaging.^7^ To overcome this problem, the large range of the detected analog signal is compressed into the effective range of the converter using a compensation circuit with a gain increasing with increasing depth (Fig. 2). Logarithmic transformation further compresses the range of the signal after an analog-to-digital conversion occurs, forming an 8-bit grayscale image to be displayed.

**Fig. 3:**
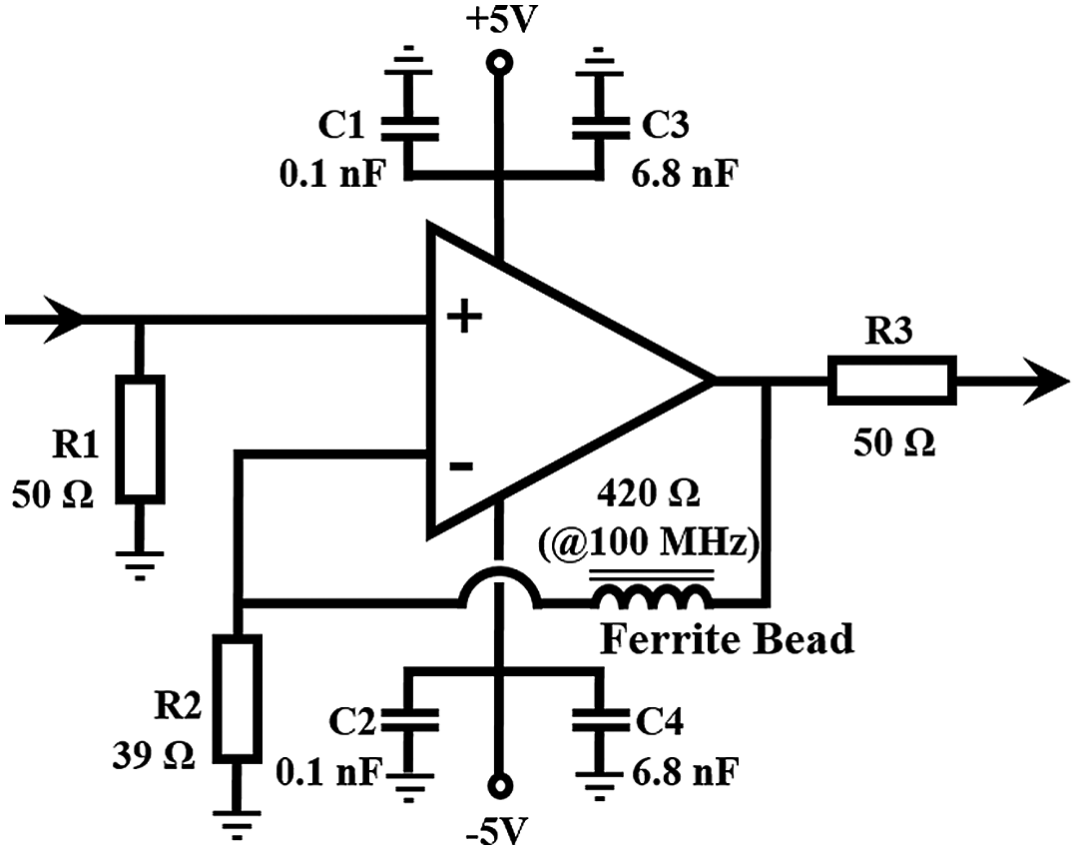
Schematic design of the frequency gain compensation amplifier.

To perform compensation, a frequency gain compensation amplifier is designed, as shown in Fig. 3. A ferrite bead (Sunlord, GZ2012D421TF) is used to replace the feedback resistor in the amplifier circuit, where the impedance of the ferrite bead increases with increasing signal frequency. According to the virtual short and virtual break characteristics of an operational amplifier, the output voltage $V_{\text{out}}$ is calculated from the input voltage $V_{\text{in}}$ as follows:

$$V_{\text{out}}=(1+R_{\mathrm{FB}}/R_{2})\times V_{\text{in}},$$ (1)

where $R_{\mathrm{FB}}$ is the impedance of the ferrite bead. Since the impedance of a ferrite bead is proportional to the frequency of the input signal, higher frequency signals are amplified with a higher gain.

## 3 Results

**Fig. 4:**
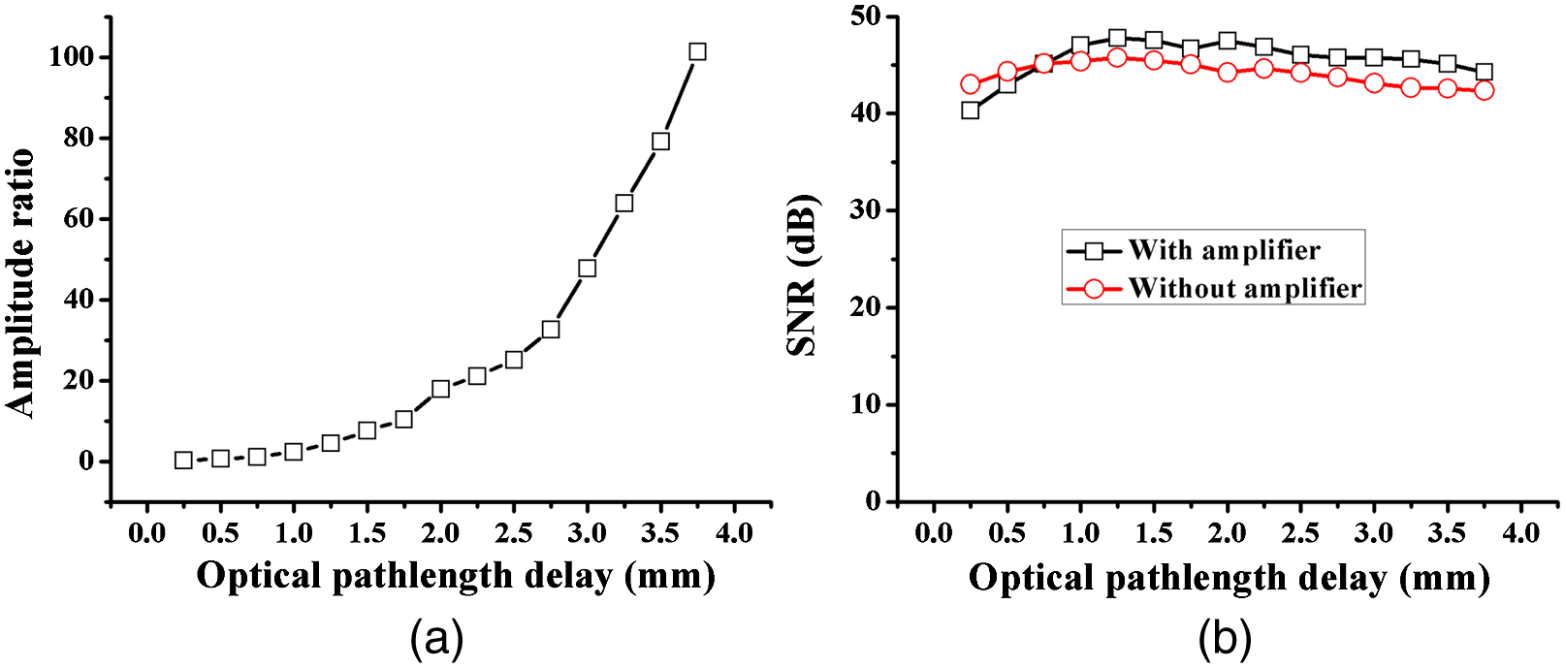
Amplitude ratio of OCT signals (a) with and without the amplifier and SNRs of OCT imaging and (b) with and without the amplifier.

A reflector is utilized as the sample to test the compensation ability of the frequency gain amplifier. Figure 4(a) shows that the amplitude ratio of the OCT signals with and without the amplifier increases from 0.26 to 101 as the path-length delay is adjusted from 0.25 to 3.75 mm, which means that the dynamic range of the OCT signals is compressed by up to 40 dB before analog-to-digital conversion. As depicted in Fig. 4(b), the signal-to-noise ratio (SNR) obtained with the amplifier decreases in the superficial layer and increases in the deep tissue compared with that without the amplifier. As the OCT image is presented in grayscale, usually, the SNR exceeds demand in the superficial layer and is insufficient in deep structures. Hence, relatively consistent quality in the whole image can be maintained with this approach. The gain curve of the amplifier can be further controlled by optimizing the design and adjusting the parameters.

**Fig. 5:**
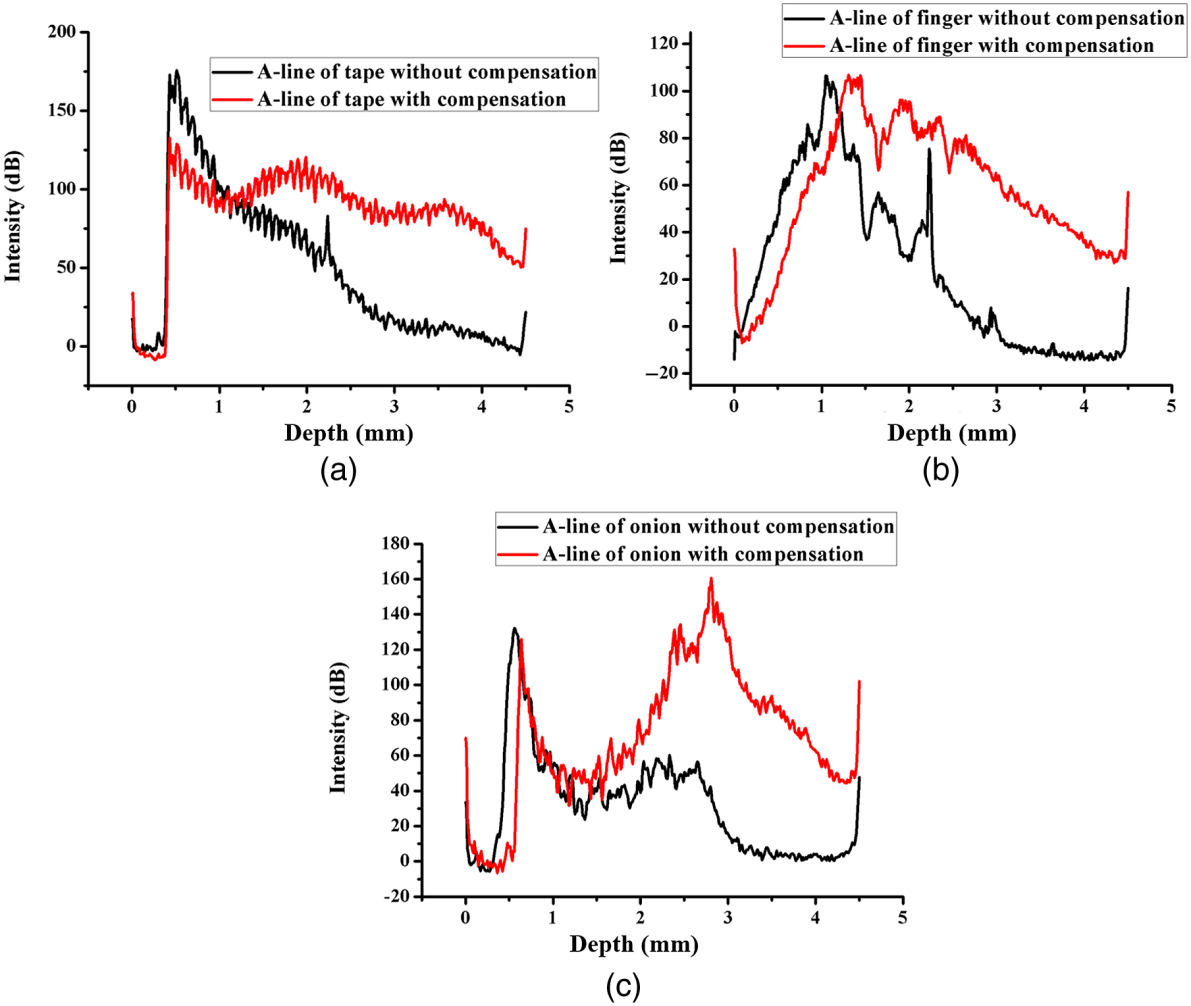
Averaged A-line intensity profiles of (a) tape, (b) finger, and (c) onion images with and without compensation.

**Fig. 6:**
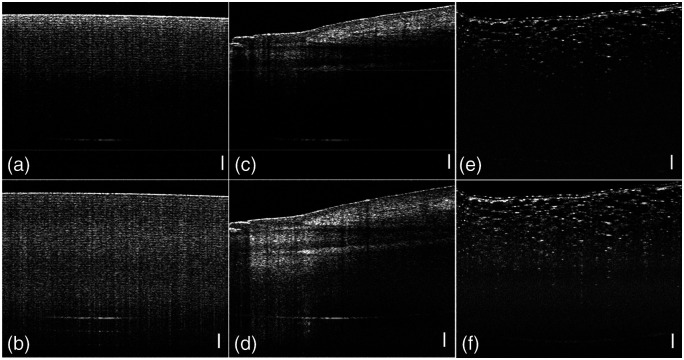
OCT images of Scotch tape (a) without and (b) with compensation, a fingernail (c) without and (d) with compensation, and onion (e) without and (f) with compensation. (scale bar: 500  μm).

In addition to testing with a mirror, we evaluated our technique by imaging three types of samples, including a roll of Scotch tape, a fingernail, and an onion skin. As shown in Fig. 5, the compensation through the frequency gain control presents a more uniform signal intensity profile. Similar to the intensity, the contrast of the OCT image before compensation also decays with increasing depth, resulting in low visibility in deep structures, as shown in Figs. 6(a), 6(c), and 6(e). In comparison, the reconstructed OCT images after compensation present a more uniform contrast through the whole image and improved visibility of the structure far below the surface, as shown in Figs. 6(b), 6(d), and 6(f).

**Fig. 7:**
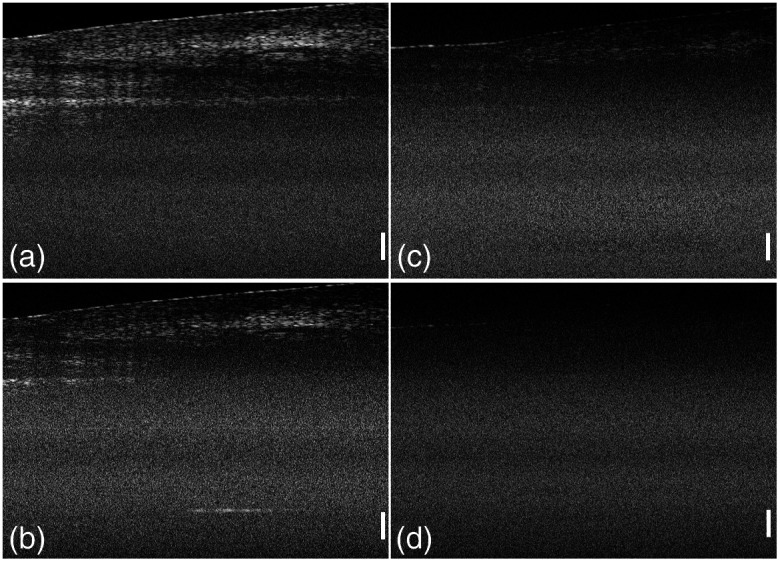
OCT images of a human fingernail under weak signal conditions. Images obtained using (a) the frequency gain compensation and (b) a digital compensation with the input light intensity attenuated to 10% and images obtained using (c) the frequency gain compensation and (d) a digital compensation with the input light intensity attenuated to 1% (scale bar: 500  μm).

The effectiveness of the compensation strategy presented in this paper is further evaluated in comparison with that of a numerical approach. A digital amplifier with a gain curve identical to the proposed compensation amplifier is used to magnify the OCT signal after the ADC. To simulate the detection capability of our method under weak signal conditions, the laser intensity coupled into the sample arm is attenuated by 90% and 99%. Figures 7(a) and 7(b) show the images of a human fingernail with the input light intensity attenuated to 10%. In Fig. 7(a), more structural information is presented with our method, whereas in Fig. 7(b), some fine structural information below the tissue surface is lost. When the input light intensity is further attenuated to 1%, all structural information is lost and could not be recovered by the numerical method, as shown in Fig. 7(d), while in Fig. 7(c), the structural information of the tissue can still be detected based on this frequency gain compensation amplifier.

## 4 Conclusion

In conclusion, we designed and fabricated a frequency gain compensation amplifier to compress the dynamic range of the electrical signal detected by the photodetector of an SSOCT system, which enables weak signals from deep structures to be acquired by the ADC and displayed with enhanced local contrast. We also experimentally evaluated the effectiveness of the technique and compared it with a numerical method. It was demonstrated that the frequency gain compensation amplifier-based approach could effectively recover the structural information lost in the acquisition process without additional software processing.
